# Promoting Students’ Well-Being and Inclusion in Schools Through Digital Technologies: Perceptions of Students, Teachers, and School Leaders in Italy Expressed Through SELFIE Piloting Activities

**DOI:** 10.3389/fpsyg.2020.01563

**Published:** 2020-07-30

**Authors:** Sabrina Panesi, Stefania Bocconi, Lucia Ferlino

**Affiliations:** Institute for Educational Technology, National Research Council of Italy, Genova, Italy

**Keywords:** inclusion, well-being, digital education, digital competence, SELFIE, DigCompOrg

## Abstract

Digital technology in its various forms is a significant component of our working environment and lifestyles. However, there is a broad difference between using digital technologies in everyday life and employing them in formal education. Digital technologies have largely untapped potential for improving education and fostering students’ well-being and inclusion at school. To bring this to fruition, systemic and coordinated actions involving the whole school community are called for. To help schools exploit the full range of opportunities digital technologies offer for learning, the European Commission has designed and implemented a self-reflection tool called SELFIE (Self-reflection on Effective Learning by Fostering Innovation through Educational Technology). Based on the DigCompOrg conceptual framework, SELFIE encompasses key aspects for effectively integrating digital technologies in school policies and practices. The present study investigates how SELFIE can also support the school community to self-reflect about students’ well-being and inclusion. In Italy, the SELFIE online questionnaire has been completed by 24,715 students, 5,690 teachers, and 1,507 school leaders, for a total of 31,912 users from 201 schools (at primary, lower secondary, and upper secondary levels) located in 10 different regions. The complementary data we have collected regarding student well-being and inclusion highlight significant differences in the perceptions on this issue reported by students, teachers, and school leaders. These findings have important implications for facilitating successful practices within the whole school community in order to promote students’ well-being and inclusion using educational technologies, as well as for planning future actions following a systemic approach.

## Introduction

### Student Well-Being and Inclusion in Schools

Over the past decade, there has been growing interest in students’ well-being, not only in relation to how it may impact on their learning, but also at policy level, examining whether and how education systems that prioritize student well-being foster positive and fulfilling life experience (Pollard and Lee, 2003). The Programme for International Student Assessment (PISA) defines well-being as the quality of students’ life, focusing on their psychological, cognitive, social, and physical capabilities. It also distinguishes between various dimensions of well-being, including life as a whole, self-related well-being, school-related well-being, and well-being out of school (OECD, 2019). This definition also emphasizes students’ well-being as an active inner process to achieve their personal and social goals (Borgonovi and Pál, 2016). Student well-being covers four discrete aspects that are nonetheless strictly corelated. The first is *cognitive well-being*, namely, successful participation in society in a variety of roles—as lifelong learners, as productive workers, as active citizens—thanks in part to their possessing the knowledge and competences required to fulfill those roles effectively. The second aspect is *psychological well-being*, namely, students’ opinion and feelings about their own lives, their educational activity, and the personal objectives they have set themselves. The third is *physical well-being*, in other words, their health level and capacity to lead a healthy lifestyle. The last facet is *social well-being*, covering relations with the family, other learners, and educators, as well as perception of the school social environment. In particular, relations with peers and educators often prove to be very strong indicators of other well-being aspects (Suldo et al., 2009; Moore et al., 2017; Littlecott et al., 2018). Investigations that Govorova et al. (2020) conducted in 35 countries within the OECD examined the range and impact on learner well-being of different factors present in learning settings; specifically, they examined measures educational institutions can take to improve learners’ perceived sense of well-being. They found that actually such measures had little substantial impact when viewed within the overall sample. Nevertheless, the authors stress the need for schools to adopt a more holistic approach and to ensure that daily educational activities take adequate account of student well-being, especially regarding the social, psychological, and emotional dimensions of the student experience.

A number of investigations such as that by de Róiste et al. (2012) highlight the considerable gains in social and emotional well-being that can be attained through learners’ more active involvement in school activities, with benefits in other areas as well, like improved learning outcomes and experiences, higher satisfaction, as well as stronger relationships and engagement levels (Kuurme and Carlsson, 2010; Løhre et al., 2010; Coombes et al., 2013). Analysis that Anderson and Graham (2016) performed of the perceived well-being levels of learners, teachers, school leaders, and support staff in a large-scale study conducted in Australia found that learners consider well-being as multi-faceted; it may comprise such aspects as student voice and regard for their positions, the exercising of rights and commanding respect. In the study, learners and school staff alike highlighted recognition of their voices and their own selves as valuable, respect-worthy members of the community as areas of particular significance for well-being. Research into the student–teacher relationship in its varied forms has revealed that teachers place special importance on discussing a wide range of matters with their students beyond study-related topics as a means of forging strong bonds and thereby contributing to well-being (Maelan et al., 2018). It should be stressed, however, that only teachers were surveyed in this case. Indeed, in much of the extant literature, “teachers” and “school staff” are treated as being synonymous, despite the burgeoning range of different support roles currently being performed by school staff members, which could well have a beneficial impact on learner well-being (van Petegem et al., 2007).

Students’ well-being at school is increasingly conceptualized not only at the individual level but also as a collective, school-wide commitment. Schools fostering the individual and collective well-being of students provide essentials for their holistic growth and development, at school and beyond. To this end, schools play a pivotal role in students’ individual and collective well-being, incorporating well-being into planning and processes, striving for excellence in teaching and learning, connecting on many levels, and helping to build trusting and respectful relationships for students to succeed. For example, the draft Curriculum for Wales 2022 (Welsh Government, 2020) seeks to embed health and well-being into the core of the new curriculum by making it one of the six “Areas of Learning Experience” for Welsh schools. It is intended to help learners understand and appreciate how the different components of health and well-being are interconnected and recognizes that good health and well-being are important to enable successful learning.

Another framework for investigating school climate and learner well-being is that formulated by the OECD (2019). This covers four areas of school experience. The first is s*afety*, pertaining to maladaptive behaviors like classroom discipline and student bullying, as well as the school’s regulations, stances, and strategies on such behavior. The second area is *teaching and learning*, comprising academic support, response and engagement, civic education and socio-emotional abilities, as well as indications of impact deriving from continuing professional development and school leadership, like school vision and teachers’ peer collaboration. The third area is *school community*, which comprises the likes of student–teacher relations, student cooperation and teamwork, respect for diversity, involvement of parents, partnerships with the local community, and the sense of involvement and belonging. The fourth area is the *institutional environment*, covering aspects like the school’s facilities and resources, availability of learning resources and digital technology, and measures of school organization like the size of the school and its classes.

Similarly, the Australian Well-being Framework for Schools (NSW Department of Education and Communities, 2015) recognizes the strong linkages between student safety, well-being, and learning outcomes, and identifies five key elements for fostering well-being within the complex, multi-dimensional world of schools. The first element is *leadership*, given that school heads and leaders are pivotal for developing a positive socio-educational environment in which all members of the school community have a sense of being included and connected, feeling both safe and respected. The second area is *inclusion*, whereby the entire school community is proactive in fostering a welcoming socio-educational culture, one in which diversity and sound, respectful relationships are valued. The third area is Student Voice, meaning that students play an active role in their learning and well-being, develop a sense of feel connectedness, and employ their social and emotional abilities to ensure they act respectfully, resiliently, and safely. The fourth area regards collaborative partnerships with families and local communities as partners as part of students’ learning, safety, and well-being. The fifth area is *support* among staff, students, and families, who work together to foster a sense of well-being and to promote positive behavior, ultimately leading to more effective teaching and learning. Hence, the Well-being Framework for Schools provides a broader understanding of well-being that encompasses multi-dimensional aspects of schools. It also conceptualizes inclusion to a degree as a different but interrelated factor contributing to well-being in the school context.

Inclusion is defined by Ainscow (2005) as the constant, ongoing quest to optimize responses to diversity, involving the abolition of obstacles to physical presence, active participation, and attainment. These factors are particularly relevant for any students facing the potential risk of being marginalized or of underachieving. In Ainscow’s view, the concept of inclusion also extends to the removal of negative responses and/or attitudes to diversity regarding a person’s race, ethnicity, gender, sexual orientation, social class, economic status, religion, first language, achievement levels, not to mention disability (Ainscow et al., 2016; Messiou, 2017). A major defining characteristic of inclusive education is response to student diversity through the deployment of learning environments and learning opportunities for all (Slee, 2018). This ensures that all learners have the potential to be an integral part of the school community and to engage actively in all facets of school life (Spratt and Florian, 2015).

In the Italian education context, Benigno et al. (2018) clearly point out that, in recent definitions of school inclusion, students’ universal access to education is combined with the pivotal role schools play in fostering a sense of collective belonging to an amicable network of individuals. Indeed, peer interaction is now seen by many as an important component of inclusion, although terms like *integration*, *participation*, and *social inclusion* are rarely defined in clear explicit terms. That notwithstanding, in much of the literature on the subject, aspects related to these fundamental concepts are considered crucial, including constructs like participation in group activities, incidence of peer interactions, perception of acceptance, and friendly relations. The study recently conducted by Sarti et al. (2019) highlights that the perception of support from others and the possibility to ask help when needed is fundamental to build social inclusive schools’ environments, especially for students with specific learning disabilities.

Moreover, children with special needs attend regular classes in Italy, from primary to secondary school, hence following the same curriculum as their peers (Zanobini et al., 2017). Despite this, problems have emerged concerning the actual degree of inclusiveness in Italy’s schools (Opertti, 2015). Principally, it has become apparent that mere attendance of students with disabilities in mainstream classes does not in itself mean that the curriculum is universally shared (Obiakor et al., 2012). Some research has demonstrated that a degree of exclusion also occurs in what are ostensibly inclusive classes, in which learners with disabilities show signs of feeling low acceptance in class (D’Alessio, 2011; Zanobini, 2013; Benigno et al., 2019).

In order to guide schools through a process of inclusive school development, the Centre for Studies on Inclusive Education has defined the *Index for Inclusion* (Booth and Ainscow, 2011), an effort intended to foster high achievement levels for all staff and students. The view of inclusion that the Index embodies is to minimize barriers to learning and participation within school policies and practices and to emphasize the student diversity as a rich resource for supporting teaching and learning. The CSIE Index is founded on the social model of disability and includes three dimensions: development of inclusive cultures, formulation of inclusive policies, and evolution in inclusive practices. Each of these is associated to a set of indicators and a number of questions. For instance, *Dimension C—Evolving inclusive practices* comprises a group of indicators that instantiate major aspects of inclusive education: all learners are encouraged to participate actively in class; they are actively engaged in their own learning; students collaborate for learning; teachers seek to foster participation and facilitate learning for all students.

### Supporting Student Well-Being and Inclusion in Schools Through Technology

In considering the matter of students’ well-being and inclusion at school, it is fundamental to understand the role and impact technology may have on these two dimensions. Evidence suggests that technologies offer opportunities for inclusive education, helping in particular to prepare learners with specific needs (related to disability, immigrant background, and socio-economic disadvantage) acquire skills that enable them to integrate into education and society as well (e.g., Benigno et al., 2019).

The European Digital Strategy (European Commission, 2020) recently announced by the European Commission highlights digital inclusion among the key priorities for the coming years. The Commission’s efforts to ensure that everybody can contribute to and benefit from the digital economy and society reflects an inclusion-driven approach through digital technologies that centers around four main pillars: (i) advancing accessible ICT solutions (design for all), (ii) developing assistive technologies enabling people with disabilities to interact, (iii) empowering citizens’ skills and digital skills to fight marginalization and social exclusion, and (iv) fostering social inclusion and participation of disadvantaged people in public, social, and economic activities. The use of digital technology provides individuals with opportunities for accessing information, managing their own learning processes, communicating with peers and mentors, and developing, repurposing, and sharing materials (Bocconi and Ott, 2013, 2014). To effectively design inclusive learning environments, teachers require specific training activities (Caruso and Ferlino, 2018), as well as to further develop a wide range of digital pedagogical competences so as to promote inclusive and personalized learning (Redecker, 2017; Bocconi and Panesi, 2018b; Caena and Redecker, 2019).

Defining the contribution that digital technologies can make in promoting inclusive socio-educational processes, Trentin (2019) proposes the *hybrid inclusive classroom model*. This entails *always-on* education opportunities for homebound students (e.g., those with chemical sensitivity illnesses), who can thereby actively contribute and take part in daily classroom life from a remote location. Hybrid inclusive classrooms unfold within dynamic hybrid spaces formed when participants use their (mobile or fixed) devices to connect online at any time, thereby integrating remote (and/or virtual) spaces and situations within the actuality of a visual/perceptual location/situation. From the learning viewpoint, hybrid classrooms exploit the *liquid nature* of digital interaction, melting the institutional rigidity that typifies schools and thereby opening up spatial–temporal and conceptual crossflows and currents (Trentin, 2017; Benigno et al., 2018). Critically, this approach also allows homebound students to maintain social relations with their peers, something that plays a central role in the development of the mind and of those social, cognitive, and meta-cognitive abilities that empower the individual to grasp and manage their inner world and well-being. Clearly, there are also significant knock-on benefits for the peers (and teachers) of homebound students, whose participation in hybrid classrooms not only impacts positively on their innate sense of inclusion but also broadens and strengthens their sense of how digital technologies can empower and shape educational processes *per se*, with potential benefits for digitally driven well-being.

Acknowledgment that *digital well-being* is intrinsic to digital competence is also a critical consideration for learning institutions (Gui et al., 2017). Authors such as Beetham (2015) argue that *digital well-being* concerns not only the supposed benefits of digital engagement but also some of its possible risks. In this view, the digital well-being of students differs substantially from that of education staff. This manifests, for example, in learners not realizing that certain online behaviors are illegal, or staff members’ stress deriving from digital work and health issues connected to digital activities.

In the Italian context, Bicocca University in Milan ran a project called “Digital Well-being – Schools” whose objective was to help educators acquire the skills needed to cooperate with students in the development of a mindful relationship with digital media and for fostering digital well-being in all areas of day-to-day life. The project deployed a training intervention that was subjected to a randomized controlled trial involving 15- to 16-year-old students from 18 high schools located in northern Italy. The outcomes corroborate the belief that fostering more aware employment of digital media can bring benefits of various kinds, one of which is everyday digital well-being. To give some examples, the test cohort began using smartphones in a less obsessive and invasive manner, scaled back their social media use, and experienced less distress related to the internet; in addition, their indices of life satisfaction and happiness increased (Gui et al., 2018).

Approaching digital well-being within education involves helping students to use digital technologies in a safe and effective manner. The need to include safety as a core element of digital education is born out by the risks to students’ individual well-being posed by phenomena like technology addiction and cyber bullying. This is supported by Mascia et al. (2020), who shed light on the multifaceted relationships that impact on the well-being of adolescents and confirmed the harm that smartphone addiction exerts on well-being and self-regulation. The European Commission’s Digital Competence Framework (DigComp 2.1) (Carretero et al., 2017) positions well-being in relation to competences connected to Safety. The framework describes protecting health and well-being as the individual’s ability to safeguard oneself and others from possible dangers and to limit risks while using technologies, including understanding the potential of technologies *for promoting social well-being and inclusion*. Seen thus, DigComp2.1 defines the social *well-being* and *social inclusion* of leaders as complementary objectives that coalesce, especially regarding the affordances and outcomes of employing digital technologies for learning (e.g., Jones and Sandford, 2019).

Similarly, at school level, the European Framework for Digitally Competent Educational Organizations (DigCompOrg) provides a comprehensive background for effectively integrating digital technologies in educational organizations (Kampylis et al., 2015; Mattar et al., 2020). This conceptual framework is the basis for the SELFIE tool^1^ that gives schools a holistic view of how students, teachers, and school leaders perceive the digital *status quo* of their policies and practices (Castaño-Muñoz et al., 2018).

### Perceptions of Students, Teachers, and School Leaders on Well-Being and Inclusion Expressed Through SELFIE

SELFIE (Self-assessment tool for digitally capable schools) is one of the 11 initiatives set out in the Digital Education Action Plan adopted by the European Commission (2018) to promote self-assessment of digital and innovative education practices in the school context. Available in the 24 official languages of the European Union, SELFIE gathers—anonymously—the views of students, teachers, and school leaders on how technology is being used in their context. In order to implement SELFIE, an analysis was conducted on several self-assessment tools of digital readiness developed and/or used in Europe (Kampylis et al., 2016), such as Opeka and Ropeka tools in Finland (Tanhua-Piiroinen and Viteli, 2017) and Digital Schools of Distinction in Ireland (O’Leary, 2018) to name a few.

With a pilot involving more than 65,000 schools’ actors in 14 countries (e.g., Kampylis et al., 2019), including Italy (Directorate-General for Education, Youth, Sport, and Culture,, 2019; Bocconi et al., in press), SELFIE encompasses elements and descriptors that may be regarded *as intrinsically linked to students’ well-being and inclusion in schools*, fostering a deep reflection that spans from *organizational responsibilities* (e.g., Leadership, Infrastructure) to *individual responsibilities* (e.g., Teaching and Learning Practices).

While some instruments embody the specific national characteristics, SELFIE is a European level initiative that provides transparency regarding schools’ digital competence, thus aiding comparison of education systems across the continent, with benefits both for peer learning and for policymaking. By helping entire learning communities (students, teachers, and school leaders) to engage in a cyclical self-reflection process, SELFIE supports schools to grasp their progress in digitally enhanced teaching and learning. SELFIE helps them plan out an ongoing development path in terms of digital strategies and praxis, and in doing so to address issues of inclusion and learner well-being.

Thus far, studies investigating SELFIE have largely concentrated on describing the tool and associated self-reflection process, and on identifying similarities and differences with similar undertakings. The outcomes of these studies reveal that SELFIE is among the very few instruments designed for comprehensive involvement of learners in the digital self-evaluation that schools conduct (Kampylis et al., 2016, 2019; Castaño-Muñoz et al., 2018). This is a fundamental characteristic of SELFIE and its contribution to promoting Student Voice, especially in identification of any potential misalignments existing between school policy/strategy level and actual teaching/learning activities.

Other research studies (Szûcs, 2019; Bocconi et al., in press) shed light on the way in which SELFIE satisfies the need to address aspects of digital innovation across the entire educational organization. Elsewhere, Jeladze and Pata (2018) demonstrate that the data generated from the SELFIE self-reflection process help schools employing digitally technologies get a better grasp of their progress in this area; this highlights the considerable variation in levels of the digital competence among different schools. Employment of SELFIE outside the school context is investigated by Broek and Buiskool (2020), who thereby shed light on the tool’s affordances for application to non-formal and informal learning.

This contribution is unique among the research studies performed thus far on SELFIE in the sense that it reveals how the personalizable questionnaire structure and content SELFIE proposes encompass key aspects in education contexts like learner well-being and inclusion, factors that are often disregarded by similar tools focusing on digital technology use. Specifically, this study is unique among the research performed into SELFIE for the following reasons:

1. it reveals that the detail encompassed within the questionnaire makes it possible to investigate critical domain-independent areas in the school context like how the employment of digital technologies impacts on learner well-being and inclusion. In this case, perceptions on the topics of well-being and inclusion were sampled from a cross-section of the school learning community undergoing SELFIE pilot testing in Italy.
2. it furthers extant research into student well-being and inclusion via digital technology use within the context of schools’ innovation vision and planning (Carretero et al., 2017; Govorova et al., 2020). There is currently a lack of published research into these issues, so the work reported here presents fresh insights.
3. the reported findings may help to inform ongoing development of SELFIE so that the tool might help users gain a better understanding of well-being and inclusion, an aspect that characterizes other frameworks (e.g., NSW Department of Education and Communities, 2015) as well as internationally adopted evaluation tools such as PISA (e.g., OECD, 2019).

### Research Question and Hypothesis

The main objective of this study is to investigate students’ well-being and inclusion through technologies within school communities, by analyzing the perception that students, teachers, and school leaders express regarding practices deployed inside the school contexts. Specifically, the research question guiding the present study is: *How do students, teachers, and school leaders perceive students’ well-being and inclusion through technologies in schools’ policies and practices, at different education levels?*

Starting from this research question, our hypotheses are as follows:

*Hypothesis 1 (H1)*: students, teachers, and school leaders perceive a relationship between well-being and inclusion in the strategies and practices of their school;
*Hypothesis 2 (H2):* students, teachers, and school leaders across education levels (primary, lower secondary, upper secondary general, upper secondary vocational) perceive students’ well-being and inclusion through technologies differently;
*Hypothesis 3 (H3):* within schools, there is a relationship between the perception of students, teachers, and school leaders on student’s well-being and inclusion through technologies at different education levels.

## Materials and Methods

### Participants

This research is part of the wider European project to pilot test an online self-assessment questionnaire called SELFIE, which deals with the perceptions that students, teachers, and school leaders have on the use of digital technologies in the school context.

A total of 201 Italian schools in 10 different regions took part in this piloting project in 2017 on a voluntary basis. Respondents to the SELFIE questionnaire comprised 24,715 students, 5,690 teachers, and 1,507 school leaders (including principal, vice-principal, subject coordinators, ICT coordinators, etc.) at different education levels (Primary, Lower Secondary, Upper Secondary general, and Upper Secondary Vocational levels), for a total of 31,912 participants (for details, see Table 1).

**TABLE 1:** Participant type for each education level.

	**Education level**				
**Users type**	**Primary**	**Lower secondary**	**Upper secondary general**	**Upper secondary vocational**	**Total**
Students	3,158	5,572	14,295	1,690	24,715
Teachers	1,651	1,212	2,424	403	5,690
School Leaders	391	420	572	124	1,507
Total	5,200	7,204	17,291	2,217	31,912

### Procedure

From the end of September to early October 2017, students, teachers, and school leaders participated in the pilot project in Italy by filling in the online SELFIE questionnaires. During the piloting phase, participants were supported by local educational authorities—or by Institutes of Educational Research – under the supervision of the SELFIE national coordinator, namely, the National Research Council of Italy’s Institute for Educational Technology (Bocconi et al., in press).

Before the piloting activities commenced, teachers acting as SELFIE coordinators (Bocconi and Panesi, 2018a) in each pilot school were fully trained to inform their local school community (i.e., school leader, teachers, and students) about the initiative and to manage the piloting process in the school.

Furthermore, the local SELFIE coordinators registered their school/s on the SELFIE platform; subsequently, the European Commission sent them an e-mail containing a link granting access to the SELFIE questionnaires. The local SELFIE coordinators then distributed the link to participants so that they could fill in the online self-assessment questionnaires.

### Measures

*The SELFIE tool* (see Castaño-Muñoz et al., 2018, for details on reliability, consistency, and validity data) has been developed on the basis of the DigCompOrg conceptual framework dedicated to the digital competence of educational organizations (Kampylis et al., 2015; see also Kampylis et al., 2016). SELFIE includes three different instantiations, respectively, devoted to the three different target users foreseen for the questionnaire: school leaders, teachers, and students. The information collected from the student questionnaires were on their use of digital technologies in the school context. The teacher questionnaires concentrated on their practices linked to school policies. The information collected from school leader questionnaires regarded policies on the use of digital technologies for learning in the school.

Overall, the three instantiations of the SELFIE questionnaire are composed of originals items (i.e., not derived or linked to other studies) organized in the following categories: (a) Core items: these were constructed according to the DigCompOrg framework and were mandatory for all respondents; (b) Attitude and belief items: these contextualize the information collected from the core items; (c) Optional items: these were constructed according to the DigCompOrg framework but schools could decide whether to include them or not in their self-reflection exercise; (d) School-specific items: these could be composed by the individual schools themselves and added to their questionnaire; (e) Vocational-specific items: these applied exclusively to vocational schools; (f) Background characteristics: questions on the school’s demographics, resources, and context (Castaño-Muñoz et al., 2018). All items were evaluated on a five-point Likert scale.

In this study, we identified a set of SELFIE original items focusing on students’ well-being and inclusion through technologies, in line with the literature (van Petegem et al., 2007; Booth and Ainscow, 2011; NSW Department of Education and Communities, 2015; Anderson and Graham, 2016; Carretero et al., 2017; OECD, 2019; Trentin, 2019). Specifically, the selected SELFIE original items (i) were common to all three respondent groups and (ii) reflected their perceptions on six components related to students’ well-being and inclusion through technologies, namely: *relationships*, *school community*, *safety*, *individual learning needs*, *active learner*, and *collaboration* (for details, see Table 2).

**TABLE 2:** SELFIE Tool: selected original items focused on well-being and inclusion.

**Components of well-being**	**SELFIE areas Castaño-Muñoz et al., 2018**	**SELFIE item ID**	**Item formulation in SELFIE questionnaire (for students, teachers, and school leaders)**
Relationships: Learners’ relations with peers and educators within the school context	1.1 Benefits and challenges are openly discussed (Area 1. Leadership)	SL_1.1 T_1.1. A S_1.3	SCHOOL LEADERS: In our school, we discuss with teachers and students the benefits and challenges of using digital technologies for teaching and learning TEACHERS: In my school, I discuss with school leaders, teachers and students the benefits and challenges of using digital technologies for learning STUDENTS: In my school, our teachers discuss with us the benefits and drawbacks of using digital technologies for learning
School community: student–teacher communications, students cooperation and teamwork, and students’ feelings about their social life	1.7 Use of different communication tools (Area 5 Infrastructure)	SL_1.7 T_1.4 S_1.5	SCHOOL LEADERS: As part of our digital strategy, we use different communication tools within and beyond the school community according to our different communication purposes and target groups TEACHERS: In my school, I use different communication tools according to the different communication purposes and target groups STUDENTS: In my school, we use digital technologies for communicating with teachers and other students
Safety: rules, attitudes and school strategies for minimizing the negative impact on students’ mental well-being potentially caused by use of technology	1.13 Students learn how to behave safely and responsibly (Areas 4 + 6 Digital competence as outcome)	SL_1.13 T_1.10 S_1.7	SCHOOL LEADERS: As part of our digital strategy, we have guidelines for students on the safe and responsible use of digital technologies TEACHERS: In my school, I teach my students how to behave in a safe and responsible way, online and offline STUDENTS: In my school, I learn how to behave in a safe and responsible way, online and offline

**Components of inclusion**	**SELFIE Areas Castaño-Muñoz et al., 2018**	**SELFIE Item ID**	**Item formulation in SELFIE questionnaire (for students, teachers, and school leaders)**

Individual learning needs: personalizing learning and addressing individual learning needs	2.5 Digital technologies are used to address individual learning needs (Area 2. Pedagogy)	SL_3.9 T_4.4 S_2.4	SCHOOL LEADERS: In our school, teachers use digital technologies to address individual learners’ needs TEACHERS: When digital technologies are used in school, it is easier to address students’ individual needs STUDENTS: In my school, I get to do special digital activities if I need extra help or if I am ahead of the class
Actively involve students: all those in the school’s learning community take an active part and play a constructive role in positive relationship building	2.3 Digital technologies are used to actively involve students (Area 2. Pedagogy)	SL_2.3 T_2.3 S_2.2	SCHOOL LEADERS: It is part of our digital strategy, to use digital technologies to actively involve students in their learning TEACHERS: As a teacher, I use digital technologies to actively involve students in their learning STUDENTS: In my school, I use digital technologies to become a more active learner
Collaboration: Students learn collaboratively	2.10 Digital technologies are used for student collaboration (Area 2. Pedagogy)	SL_2.10 T_2.10 S_5.5	SCHOOL LEADERS: It is part of our digital strategy, that students collaborate using digital technologies TEACHERS: As a teacher, I use digital technologies to help students collaborate with each other STUDENTS: In my school, my classmates and I help each other when we have problems with digital technologies

### Statistics Analysis

To conduct qualitative investigation of the mean perceptions of students, teachers, and school leaders about items dedicated to students’ well-being and inclusion through technologies, descriptive statistics on scores related to those items were calculated. Following the literature (Könings et al., 2014), ANOVAs were not conducted to compare the means from the three groups (students, teachers, school leaders) because, in answering the questionnaire, each group was called upon to focus on different aspects: students on their use of digital technologies in the school context; teachers on their practices linked to school policies; and school leaders on policies related to the use of digital technologies for learning in the school (Castaño-Muñoz et al., 2018). For this reason, the literature stresses that it is not enough to compare only mean perception scores of different stakeholder groups (Könings et al., 2014). The relations among the scores related to the items focused on students’ well-being and inclusion for each user group (students, teachers, and school leaders) were investigated with bivariate correlations. A series of confirmatory factor analyses (CFAs), based on covariance matrices, were conducted to verify the latent structure of the perception of students’ well-being and inclusion for each user group.

Specifically, we conducted the CFA based on different theoretical models: (1) a single factor model, consistent with DigComp2.1 (Competence area “Safety”, skill “4.2 Protecting health and well-being”) (Carretero et al., 2017); (2) a two-factor model, consistent with The Well-being Framework for Schools (NSW Department of Education and Communities, 2015), where inclusions are seen as a different but interrelated factor contributing to well-being in the school context. To conduct CFA, EQS 6.1 software has been used (Bentler, 2006).

Multiple fit indices were considered to compare models: chi-square (χ^2^), the goodness-of-fit index (GFI), the adjusted goodness-of-fit index (AGFI), comparative fit index (CFI), the root mean square error of approximation (RMSEA), the standardized root mean squared residual (SRMR), and the Akaike information criterion (AIC). Chi-square was used to evaluate the appropriateness of the CFA model; non-significant values indicated a minor difference between the covariance matrix generated by the model and the observed matrix and, thus, an acceptable fit. GFI values of >0.95 can be considered a good fit and >0.90 can be considered an acceptable fit (Schermelleh-Engel et al., 2003). An AGFI index of >0.90 is indicative of good fit, while values >0.85 may be considered as an acceptable fit (Schermelleh-Engel et al., 2003). CFI values of >0.97 can be considered a good fit (Schermelleh-Engel et al., 2003), and >0.95 can be considered an acceptable fit (Schermelleh-Engel et al., 2003). RMSEA levels of <0.05 indicate a good fit and acceptable if <0.08 (Kline, 2005). SRMR <0.05 represents a good fit and <0.10 is acceptable (Schermelleh-Engel et al., 2003). AIC is used to compare models: the model with the lowest AIC values is to be preferred.

Furthermore, in the two-factor CFA model based on the theoretical well-being framework for Schools (NSW Department of Education and Communities, 2015), for each of the three samples (students, teachers and school leaders) and each of the two factors measured, we calculated McDonald’s omega, composite reliability (CR), average variance extracted (AVE), discriminant validity, and convergent validity with R package (Rosseel, 2012; Jorgensen et al., 2019), also in line with Zhang and Yuan (2016). First, McDonald’s omega (McDonald, 1970, 1999) was used to measure internal consistency reliability; values considered acceptable were those above 0.70 (Viladrich et al., 2017). Furthermore, in an effort to gauge construct validity, we employed (a) *convergent validity*, namely, confidence that the adopted indicators had accurately measured a trait, and (b) *discriminant validity*, namely, the extent to which trait measurements are not related (Campbell and Fiske, 1959; Jöreskog, 1969). To determine convergent validity, we referred to the criterion proposed by Fornell and Larcker (1981), a commonly adopted approach for evaluating the level of variance that a model’s latent variables may share. According to this criterion, the convergent validity of the measurement model can be assessed by the AVE and CR. AVE measures the level of variance captured by a construct versus the level due to measurement error, and values above 0.70 are considered very good, whereas the values of 0.50 are acceptable. CR is a less biased estimate of reliability than Cronbach’s alpha; the acceptable value of CR is 0.70 and above. According to Fornell and Larcker (1981), discriminant validity can be assessed by comparing the amount of the variance capture by the construct and the shared variance with other constructs. Thus, the levels of square root of the AVE for each construct should be greater than the correlation involving the constructs.

Based on the CFA results, composite scores were calculated as the mean of the well-being and inclusion *z* score to represent the two latent dimensions. For each user group (students, teachers, and school leaders), ANOVAs were conducted with the composite well-being and inclusion scores as dependent variables, and education level group membership (primary, lower secondary, upper secondary general, and upper secondary vocational) as the between-subject variable to explore group differences in the well-being and inclusion components. Finally, for each school, the average scores expressed, respectively, by students, teachers, and school leaders on each item were calculated. For each type of participant (students, teachers, and school leaders), composite scores were calculated as the mean of the well-being and inclusion *z* score to represent the two latent dimensions. Bivariate correlations among these scores were conducted separately for each education level.

## Results

Descriptive statistics results for the three groups (students, teachers, and school leaders) concerning the items focused on students’ well-being and inclusion through technologies are shown in Table 3. Overall, the mean scores of the participants’ perspective for each item showed teachers as the group that expressed the most negative reaction, with the exception of two items (“Use of different communication tools” and “Digital technologies are used to address individual learning needs”), where students expressed a more negative perspective.

**TABLE 3:** Descriptive statistics.

	**Students**		**Teachers**		**School leaders**	
	***M***	***SD***	***M***	***SD***	***M***	***SD***
Benefits and challenges are openly discussed	3.21	1.21	2.86	1.12	3.28	0.99
Use of different communication tools	3.25	1.94	3.40	1.01	3.68	0.94
Students learn how to behave safely and responsibly	3.48	1.24	3.43	1.08	3.69	0.94
Digital technologies are used to address individual learning needs	2.83	1.30	3.60	0.90	3.38	0.89
Digital technologies are used to actively involve students	3.46	1.11	3.36	0.96	3.64	0.84
Digital technologies are used for student collaboration	3.71	1.13	3.12	1.08	3.44	0.97
						

Table 4 reports the correlations among SELFIE items focusing on students’ well-being and inclusion for the three participant groups: students, teachers, and school leaders. The findings showed reasonable correlations among all items for students (*r* = 0.194 to *r* = 0.447; all *p* < 0.001), teachers (*r* = 0.266 to *r* = 0.621; all *p* < 0.001), and school leaders (*r* = 0.392 to *r* = 0.651; all *p* < 0.001). Particularly strong correlations emerged for the school leader group. Furthermore, for all groups, the three scores related to the answers to items focused on students’ well-being showed reasonable correlations with the three scores related to answers to the items focused on inclusion (students: *r* = 0.212 to *r* = 0.259; all *p* < 0.001; teachers: *r* = 0.266 to *r* = 0.422; all *p* < 0.001; school leaders: *r* = 0.402 to *r* = 0.508; all *p* < 0.001).

**TABLE 4:** Zero-order correlations between scores related to the items focused on students’ well-being and inclusion (separately for students, teachers, and school leaders).

	**1**	**2**	**3**	**4**	**5**	**6**
**Students**						
Benefits and challenges are openly discussed		0.207***	0.447***	0.212***	0.259***	0.257***
Use of different communication tools			0.191***	0.212***	0.259***	0.257***
Students learn how to behave safely and responsibly				0.233***	0.232***	0.230***
Digital technologies are used to address individual learning needs					0.327***	0.263***
Digital technologies are used to actively involve students						0.194***
Digital technologies are used for student collaboration						
**Teachers**						
Benefits and challenges are openly discussed		0.381***	0.402***	0.280***	0.393***	0.425***
Use of different communication tools			0.460***	0.302***	0.422***	0.412***
Students learn how to behave safely and responsibly				0.266***	0.398***	0.420***
Digital technologies are used to address individual learning needs					0.367***	0.324***
Digital technologies are used to actively involve students						0.621***
Digital technologies are used for student collaboration						
**School leaders**						
Benefits and challenges are openly discussed		0.392***	0.452***	0.455***	0.508***	0.462***
Use of different communication tools			0.462***	0.428***	0.464***	0.402***
Students learn how to behave safely and responsibly				0.462***	0.478***	0.430***
Digital technologies are used to address individual learning needs					0.547***	0.497**
Digital technologies are used to actively involve students						0.651***
Digital technologies are used for student collaboration						

*p < 0.05; **p < 0.01; ***p < 0.001.

Based on these last findings, we also performed a set of CFAs to determine the latent structure of students’ well-being and inclusion for students, teachers and school leaders. For each group (students, teachers, and school leaders) two alternative models were tested. Model A assumed that all scores loaded on a single factor. In model B, the three well-being scores loaded on one factor and the three inclusion scores loaded on the other factor. Table 5 summarizes the fit indices for these models. All models showed at least an acceptable fit to the data. For each of the three groups involved, the lowest AIC, RMSEA, and SRMR, and the highest GFI, AGFI, and CFI were found for models B (factor 1: well-being; factor 2: inclusion) Therefore, models B provided the best fit.

**TABLE 5:** Fit indices of the hypothesized models (separately for students, teachers and school leaders).

	**Models**	**Factors**	**χ^2^**	**df**	***P***	**RMSEA**	**SRMR**	**GFI**	**AGFI**	**CFI**	**AIC**
Students	A	1 factor: Well-being/inclusion	1,769.508	9	0.000	0.089	0.042	0.975	0.942	0.910	1,751.508
		2 factors	1,237.160	8	0.000	0.079	0.041	0.983	0.957	0.937	1,221.160
	B	(1) Well-being;									
		(2) Inclusion									
Teachers	A	1 factor: Well-being/inclusion	439.839	9	0.000	0.092	0.036	0.973	0.937	0.952	421.839
		2 factors	120.433	8	0.000	0.050	0.021	0.993	0.982	0.987	104.433
	B	(1) Well-being;									
		(2) Inclusion									
School Leaders	A	1 factor: Well-being/inclusion	90.980	9	0.000	0.078	0.029	0.979	0.950	0.974	72.978
		2 factors	51.496	8	0.000	0.060	0.023	0.988	0.969	0.986	35.496
	B	(1) Well-being;									
		(2) Inclusion									

The fit of nested models can be compared by subtracting the χ^2^ value of the less restricted model. Following previous research (e.g., Miyake et al., 2000; Panesi and Morra, 2020), in this study we also compare models A and B in this direct way. This is because positing one factor is mathematically equivalent to fixing at 1 the value of the parameter phi that represents the correlation between the two factors posited in any of the two-factor models. The fit of model B was significantly better than the fit of model A for students [χ^2^diff(1) = 532.348], teachers [χ^2^diff(1) = 319.406], and school leaders [χ^2^diff(1) = 39.484].

**FIGURE 1:**
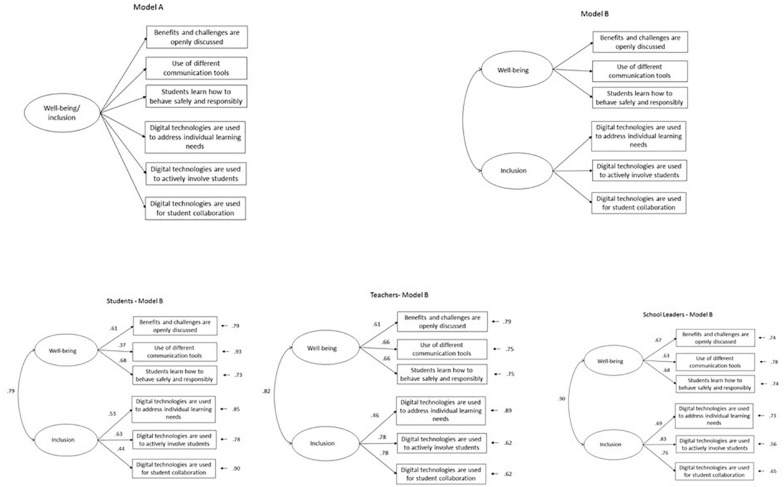
Well-being and inclusion models. Models B are the endorsed models, respectively, for students, teachers, and school leaders (standardized parameters are reported).

It should also be noted that, in the endorsed models, the estimated correlations between the two latent variables were quite large for students (phi = 0.79), teachers (phi = 0.82), and school leaders (phi = 0.90) (see Figure 1), and these findings are in line with the Well-being Framework for Schools (NSW Department of Education and Communities, 2015).

Furthermore, concerning the two-factor model (Model B), Table 6 shows, for each of the three users (students, teachers, and school leaders), the reliability of the two constructs (factor 1: well-being; factor 2: inclusion) as measured by McDonald’s omega, AVE, CR, and the square root of the AVE, to also investigate convergent and discriminant validity.

**TABLE 6:** The two-factor CFA model, for each of the three samples’ McDonald’s omega, AVE, CR, and the square root of the AVE.

	**McDonald’s omega**	**AVE**	**CR**	**√AVE**	***phi***
**Students**					
Well-being	0.57	0.32	0.71	0.56	0.79
Inclusion	0.55	0.29	0.71	0.54	
**Teachers**					
Well-being	0.67	0.40	0.82	0.63	0.82
Inclusion	0.71	0.47	0.95	0.68	
**School leaders**					
Well-Being	0.70	0.44	0.84	0.66	0.90
Inclusion	0.75	0.50	0.91	0.71	

*Internal validity (McDonald’s omega, >0.70); convergent validity: AVE (>0.50) and CR (>0.70); discriminant validity (comparison of AVE squared root > phi, correlation score of pair of latent variables).*

Concerning *internal reliability*, for the school leaders’ group, McDonald’s omega met the acceptable level for both well-being and inclusion (factor 1:0.70; factor 2:0.75), while for the teachers’ group, McDonald’s omega was slightly lower than the acceptable value (0.67) for well-being and above the threshold (0.71) for inclusion. Conversely, for the students, McDonald’s omega fell below the acceptable value for both factors (factor 1:0.57; factor 2:0.55). Therefore, reliability indexed by McDonald’s omega is sufficient for well-being and inclusion factors, with the exception of students. It should be noted that these findings mimic the characteristics of the SELFIE instrument, where school leaders and teachers provide, respectively, the perspective on the two factors (well-being and inclusion) in schools’ policy and practice, while students’ perspective falls in between the two layers, hence reasonably having a more limited perception of both.

Regarding the *convergent validity*, the CR for both well-being and inclusion surpassed the 0.70 threshold for all the three users. The AVE reached the recommended level of 0.50 in factor 2—inclusion for the school leaders, except for teachers and students. AVE values also scored below the threshold in factor 1—well-being for all the three actors. Although the AVE acceptable minimum cutoff point is 0.50, convergent validity may still be considered adequate because all latent factors had CR values above 0.70 (Malhotra and Dash, 2011). According to Fornell and Larcker (1981), convergent validity can be established via CR alone because AVE is a more conservative measure and relatively strict compared to CR. Likewise, other authors (Angel et al., 2019; Ibrahim et al., 2019; Shaakumeni and Csapoì, 2019) also insisted that latent variables’ AVE can fall below 0.50 if its CR is satisfactory.

Finally, concerning *discriminant validity*, the square root of the AVE values for both factor 1—well-being and factor 2—inclusion did not exceed the correlation between the two constructs for all the three groups as required by Fornell and Larcker (1981). It is important to note, however, that discriminant validity is not exclusively an empirical means to validate a model (Farrell, 2010). Theoretical foundations and arguments should provide reasons for constructs correlating or not (Bollen and Lennox, 1991), as concepts are partly defined by their relationships with other concepts in a conceptual network (Henseler et al., 2015). Therefore, failure to establish discriminant validity between the two constructs (well-being and inclusion) does not necessarily imply that the underlying concepts are identical, especially when research studies continued support for conceptualizing these two constructs as separated but interrelated (NSW Department of Education and Communities, 2015; OECD, 2019).

Based on the CFA results, for each group (students, teachers, and school leaders), two composite scores representing well-being and inclusion were calculated as the mean of the *z* scores, as follows: (A) for well-being: the *z* score average of the answers to the items (1) Benefits and challenges are openly discussed; (2) Use of different communication tools; (3) Students learn how to behave safely and responsibly; (B) for inclusion: the *z* score average of answers to the items (1) Digital technologies are used to address individual learning needs; (2) Digital technologies are used to actively involve students; (3) Digital technologies are used for student collaboration.

The results of ANOVAs conducted with the two composite well-being and inclusion measures as dependent variables and education level group membership (primary, lower secondary, upper secondary general, and upper secondary vocational) as the between-subject variable showed that for students and teachers, differences between education level groups emerged in both well-being and inclusion. In particular, differences were found among students of different education level groups. For the well-being component, the lower secondary group returned a more positive perception than all the others, followed by the primary group and the upper secondary vocational group. For the inclusion component, the primary group returned a more positive perception than all the others, followed by the lower secondary group and the upper secondary vocational group (for details, see Table 7). Concerning teachers, the primary group returned lower scores than other groups in both well-being and inclusion. For inclusion only, differences between the lower secondary group and the upper secondary general group and between the upper secondary general group and the upper secondary vocational group emerged. No differences were found in either the well-being or inclusion components among school leaders of any education level group.

**TABLE 7:** Descriptive statistics, ANOVA, and *post hoc* (Bonferroni).

		**Students**						**Teachers**						**School leaders**					
	**Education level**	**Descriptive statistics**		**ANOVA**		***Post hoc* (Bonferroni)**		**Descriptive statistics**		**ANOVA**		***Post hoc* (Bonferroni)**		**Descriptive statistics**		**ANOVA**		***Post hoc* (Bonferroni)**	
		***M***	***SD***	***F***	***Sig***	**Comparisons**	***Sig.***	***M***	***SD***	***F***	***Sig***	**Comparisons**	***Sig.***	***M***	***SD***	***F***	***Sig***	**Comparisons**	***Sig.***
Well-	Primary	0.223	0.713	550.803	0.000	*P* < LS	0.002	−0.115	0.835	15.679	0.000	*P* < LS	0.000	0.018	0.792	2.474	0.060	*P* = LS	1.000
being	Lower secondary	0.285	0.724			*P* > USG	0.000	0.068	0.812			*P* < USG	0.000	0.060	0.788			*P* = USG	1.000
	Upper secondary general	−0.161	0.802			*P* > USV	0.000	0.044	0.833			*P* < USV	0.072	−0.020	0.898			*P* = USV	0.211
	Upper secondary vocational	0.005	0.742			LS > USG	0.000	0.000	0.792			LS = USG	1.000	−0.164	0.879			LS = USG	0.815
						LS > USV	0.000					LS = USV	0.936					LS = USV	0.054
						USG < USV	0.000					USG = USV	1.000					USG = USV	0.503
Inclusion	Primary	0.168	0.743	132.916	0.000	*P* > LS	0.001	−0.223	0.888	59.365	0.000	*P* < LS	0.000	−0.035	0.820	1.637	0.179	*P* = LS	1.000
	Lower secondary	0.102	0.783			*P* > USG	0.000	0.009	0.870			*P* < USG	0.000	−0.021	0.882			*P* = USG	0.644
	Upper secondary general	−0.079	0.769			*P* > USV	0.000	0.149	0.873			*P* < USV	0.000	0.061	0.976			*P* = USV	1.000
	Upper secondary vocational	0.016	0.771			LS > USG	0.000	−0.008	0.854			LS < USG	0.000	−0.099	0.917			LS = USG	0.926
						LS > USV	0.000					LS = USV	1.000					LS = USV	1.000
						USG < USV	0.000					USG > USV	0.005					USG = USV	0.443

Finally, average school scores were calculated for students, teachers, and school leaders on each item. Subsequently, composite scores were calculated as the mean of the well-being and inclusion *z* score to represent the two latent dimensions, respectively, for students, teachers, and school leaders. Table 8 reports the correlations among the two composite well-being and inclusion measures that reflect students’, teachers’, and school leaders’ perception at different education levels. Overall, the findings showed strong correlations among teachers’ and school leaders’ perception on both well-being and inclusion at all education levels (*r* = 0.476 to *r* = 0.622, all *p* < 0.001), except for upper secondary vocational level.

**TABLE 8:** Zero-order correlations within schools on well-being and inclusion (separately for education levels).

	**1**	**2**	**3**	**4**	**5**	**6**
**Primary (School *n* = 59)**						
1. Students’ well-being		0.392**	0.407**	0.357**	0.237	0.309*
2. Students’ inclusion			0.418**	0.495***	0.279*	0.423**
3.Teachers’ well-being				0.806***	0.540***	0.542***
4.Teachers’ inclusion					0.519***	0.476***
5.School leaders’ well-being						0.665***
6. School leaders’ inclusion						
**Lower secondary (School *n* = 65)**						
1.Students’ well-being		0.516***	0.432***	0.380**	0.396**	0.272*
2.Students’ inclusion			0.494***	0.652***	0.480***	0.525***
3.Teachers’ well-being				0.796***	0.519***	0.491***
4.Teachers’ inclusion					0.523***	0.511***
5.School leaders’ well-being						0.791***
6. School leaders’ inclusion						
**Upper secondary general (School *n* = 62)**						
1.Students’ well-being		0.679***	0.650***	0.494***	0.461***	0.351**
2.Students’ inclusion			0.531***	0.464***	0.412**	0.453***
3.Teachers’ well-being				0.901***	0.621***	0.555***
4.Teachers’ inclusion					0.622***	0.587***
5.School leaders’ well-being						0.849***
6. School leaders’ inclusion						
**Upper secondary vocational (School *n* = 14)**						
1.Students’ well-being		0.434	0.635*	0.246	0.224	0.348
2.Students’ inclusion			0.284	0.390	0.049	0.143
3.Teachers’ well-being				0.757**	0.338	0.521
4.Teachers’ inclusion					0.114	0.252
5.School leaders’ well-being						0.348
6. School leaders’ inclusion						

*^∗^*p* < 0.05; ^∗∗^*p* < 0.01; and ^∗∗∗^*p* < 0.001.*

Concerning students’ perception on well-being and inclusion, some particular results may be observed at school level. In primary schools, the highest correlations with teachers’ perception on well-being and inclusion (*r* = 0.357 to *r* = 0.495, all *p* < 0.01) emerged, but also reasonable correlations with school leaders’ perception on inclusion and an acceptable correlation between students’ perception on inclusion and school leaders’ perception on well-being emerged (all *p* < 0.05). In lower secondary schools, all scores correlated significantly (all *p* < 0.01), and in particular, students’ perception on well-being correlated highest with teachers’ perception on well-being (*r* = 0.432, *p* < 0.001) and subsequently with school leaders’ perception on well-being (*r* = 0.396, *p* < 0.01) and students’ perception on inclusion correlated highest with teachers’ perception on inclusion (*r* = 0.652, *p* < 0.001) and subsequently with school leaders’ perception on inclusion (*r* = 0.525, *p* < 0.001). In upper secondary general schools, high correlations with teachers’ perception on well-being and inclusion emerged (all *p* < 0.001), followed by significant correlations with school leaders’ perception on well-being and inclusion (all *p* < 0.01). In upper secondary vocational, only a correlation between students’ perception on well-being and teachers’ perception on well-being (*r* = 635, *p* < 0.05) emerged.

## Discussion and Conclusion

The findings of this study highlight the strong relationship between students’ well-being and inclusion through technologies perceived by students, teachers, and school leaders in schools’ policies and practices. In addition, depending on the education level, the perceptions of the three actors emerge as different and correlated.

A number of studies in the literature highlight a strong relationship between well-being and inclusion in schools (e.g., NSW Department of Education and Communities, 2015), including through technologies (Carretero et al., 2017; OECD, 2019). To the best of our knowledge, however, few works explore the relationship among the perceptions of students, teachers, and school leaders on students’ well-being and inclusion through technologies specifically in schools’ policies and practices in order to effectively meet the needs of students.

This study attempted to identify how students, teachers, and school leaders perceive students’ well-being and inclusion through technologies in schools’ policies and practices and has highlighted some interesting insights in relation to the formulated hypothesis.

In particular, concerning the perception of students, teachers, and school leaders about the relationship between well-being and inclusion in the policies and practices of their school (*H1*), the findings indicate that (i) the different components of well-being (*relationships*, *school community*, *safety*) and of inclusion (*individual learning needs*, *active learning*, and *collaboration*) are perceived by all the three actors as associated; in particular, the highest correlations emerged in school leaders’ perceptions. (ii) Well-being and inclusion are perceived by all the three actors as two separated but strongly correlated factors, mostly by school leaders. These findings confirm Hypothesis 1 and are in line with consolidated international frameworks where inclusion is seen as a different but interrelated factor contributing to well-being in the school context (NSW Department of Education and Communities, 2015; OECD, 2019). The fact that the strongest correlation between components of well-being and inclusion emerged in school leaders’ perceptions could be explained either by the systemic and holistic point of view that characterize school leaders’ and by their commitment to ensure compliance with mandated education policy (Tosh and Doss, 2020).

Depending on the education level, students, teachers, and school leaders perceive students’ well-being and inclusion through technologies differently (*H2*). Specifically, the most positive perception on well-being was expressed by students in lower secondary, followed by students in primary, in upper secondary vocational, and, lastly, in upper secondary general (e.g., licei and technical schools). On the other hand, the most positive perception on inclusion was expressed by students in primary school, followed by students in lower secondary and upper secondary vocational. Once again, the most negative perception was expressed by students in upper secondary general. These findings are in line with recent PISA results (OECD, 2019), indicating that around one-third of 15-year-old students were not satisfied with their lives (both in and out of school). Concerning teachers, few differences emerged at various school levels. The most negative perception regarding both inclusion and well-being emerge from primary school teachers. This finding is line with the literature, which shows primary teachers’ need for collaboration, shared responsibilities, common planning time, and professional development to foster inclusion and well-being through technologies (Sam et al., 2015; Peacock, 2016; Manrique et al., 2019). Regarding school leaders, no significant differences emerge at different education levels. These findings partially confirm Hypothesis 2. As argued by Castaño-Muñoz et al. (2018), school leaders’ perceptions are unlikely to vary significantly among education levels as they reflect the policy and strategy dimension of the school context (Tuytens and Devos, 2010). By contrast, students’ and teachers’ perceptions reflect classroom practices, which are most likely affected by factors related to the education level.

Concerning our third hypothesis, within schools, a relationship between the perception of students, teachers, and school leaders on student’s well-being and inclusion through technologies emerged at different education levels (*H3*). The findings highlight that (i) teachers and school leaders’ perceptions are strongly correlated at all education levels, except for iVET; this could result from the limited size of the sample of iVET schools involved in the study. (ii) Students’ perceptions most closely correlate with teachers’ perceptions with respect to school leaders’ perceptions at all education levels, except for iVET. These findings confirm Hypothesis 3 and are in line with the literature that highlights relationships between students, teachers, and school leaders’ perception (e.g., Castaño-Muñoz et al., 2018) on students’ well-being and inclusion through technologies (Walker and Logan, 2009; Soutter et al., 2014; Ovbiagbonhia et al., 2019). In particular, the strong relationships that emerged between students and teachers, and between teachers and school leaders highlight the importance of considering the perceptions of all the three main actors in the school community to promote well-being and inclusion (Kampylis et al., 2015; OECD, 2019). This is in line with the evidence that school climate is as an important factor to be considered to improve engagement in school activities, but it is effective only when its influence can modify the well-being experience of the students (Lombardi et al., 2019).

A major limitation of the present research study regards the low degree of reliability and validity of the SELFIE tool and process for the specific purpose of assessing the school community’s (students’, teachers’, and school leaders’) perceptions of how digital technology use affects students’ well-being and inclusion. Indeed, while an adequate level of convergent validity was returned for all three participant groups, the internal reliability of both well-being and inclusion factors was insufficient for the student group, as was the well-being factor related to the teachers’ group. Likewise, the discriminant validity level was inadequate across the board, i.e., for both factors in relation to all the user groups. These results can be attributed to two main reasons: (i) as a tool expressly designed to support schools’ self-evaluation of the use of digital technologies for teaching and learning *per se*, SELFIE’s focus on well-being and inclusion through technology is quite limited in scope; (ii) this aligns epistemologically with the theoretical frameworks (NSW Department of Education and Communities, 2015; OECD, 2019) underpinning the two-factor CFA models, which in fact treat well-being and inclusion as distinct but tightly intertwined factors.

Those endeavoring to repeat the research study reported here are advised to include external variables as a means of validating our theoretical assumption, mentioned above, that well-being and inclusion are distinct factors that in fact are tightly intertwined. In addition, they could look in greater detail on SELFIE’s limited scope for gauging school-wide perceptions on the relations between digital technology use and learners’ well-being and inclusion. One possible step in this direction would be to consider employing other tools as well, such as PISA (OECD, 2019) for well-being and the Index for Inclusion (Booth and Ainscow, 2011) for inclusion. Another constructive step could be to engage other stakeholder groups—especially parents—in the SELFIE process, especially to increase the tool’s scope to address digital technology use relations with well-being and inclusion. Clearly, systematic field studies are required to gain a firmer grasp of how digital technologies can be employed for positive, school-level practices that support well-being and inclusion.

In conclusion, the findings presented here highlight significant factors regarding the relations between digital technology use and students’ well-being and inclusion, factors that ought to be addressed so as to satisfy the needs of students in an effective manner.

Practical implication to improve schools’ digital policies and practices can be drawn from our findings. In particular, the strong relationship between students and teachers’ perceptions can guide teachers’ cooperation, by exchanging ideas and sharing best practices on how to provide extra help or giving students opportunities to express their ideas in relation to well-being and inclusion through technologies. In addition, the relationship of school leaders’ perceptions with both teachers and students can contribute to design consistent schools’ policies, building trusting relationships with teachers and students, and to offer enriching activities for effectively responding to students’ needs. This is in line with recent empirical studies on well-being (Lombardi et al., 2019) that emphasize the importance of integrating socio-emotional development in schools’ daily practice (Burns and Gottschalk, 2019). Such type of school-level interventions, which have emerged to be more successful in some European countries including Italy (Govorova et al., 2020), encompasses key components of well-being and inclusion through technologies such as *relationships*, *school community*, *safety*, *individual learning needs*, *active learning*, and *collaboration*, explored in the SELFIE self-reflection process (Kampylis et al., 2016, 2019). Similarly, research currently being performed in the EC ERASMUS+SHERPA project (ErasmusPlus, 2020) includes the design and testing of a pedagogical innovation toolkit that complements and operationalizes schools’ SELFIE self-reflection on digital technology use. The kit will scaffold individual school’s efforts to formulate sound policies and practices in this area, and in doing so will provide adequate scope for addressing aspects related to students’ well-being and inclusion. The focus on the school-wide environment will help in identifying variables in the school climate that impact on students’ well-being and inclusion, thereby also enriching the SELFIE tool and process itself.

## Data Availability Statement

The datasets generated for this study are available on request to the corresponding author.

## Ethics Statement

Ethical review and approval was not required for this study involving human participants in accordance with the local legislation and institutional requirements. All answers provided through SELFIE were anonymous. Individual students, teachers, school leaders or other staff members replying to the questions and statements were not identified personally.

## Disclaimer

The views expressed in this article are purely those of the authors and should not be regarded as the official position of the European Commission.

## Author Contributions

SP contributed to the organization and data collection, performed statistical analyses, participated in the interpretation of results, and contributed to writing the manuscript, in particular the sections Materials and Methods, Results, and Discussion and Conclusion. SB has organized and supervised data collection, conducted the literature review, participated in interpretation of the results, and contributed to writing the manuscript, in particular the sections Introduction and Discussion and Conclusion. LF has contributed to the literature review and provided feedback on the manuscript. All authors read and approved the submitted version.

## Conflict of Interest

The authors declare that the research was conducted in the absence of any commercial or financial relationships that could be construed as a potential conflict of interest.

## Footnotes

1 https://ec.europa.eu/education/schools-go-digital_en
